# Characterizing the Immune Microenvironment and Neoantigen Landscape of Hürthle Cell Carcinoma to Identify Potential Immunologic Vulnerabilities

**DOI:** 10.1158/2767-9764.CRC-23-0120

**Published:** 2023-07-31

**Authors:** Ian Ganly, Fengshen Kuo, Vladimir Makarov, Yiyu Dong, Ronald Ghossein, Bin Xu, Luc G.T. Morris, Timothy A. Chan

**Affiliations:** 1 Human Oncology and Pathology Program, Memorial Sloan Kettering Cancer Center, New York, New York.; 2 Department of Surgery, Memorial Sloan Kettering Cancer Center, New York, New York.; 3 Center for Immunotherapy and Precision Immuno-Oncology, Cleveland Clinic, Cleveland, Ohio.; 4 Department of Pathology, Memorial Sloan Kettering Cancer Center, New York, New York.

## Abstract

Hürthle cell carcinoma (HCC) is a rare type of thyroid cancer with high rates of distant metastasis and recurrence. Along with the scarcity of effective systemic therapies for HCC, these factors contribute to poor clinical outcomes. The immunologic features of HCC are poorly defined and response rates with immune checkpoint blockade have not been reported. A more comprehensive understanding of the immune landscape and factors that predict response to checkpoint inhibitors is needed.

We performed RNA sequencing on 40 tumors to characterize the neoantigen landscape and immune microenvironment of HCC. We analyzed transcriptomic profiles, tumor-infiltrating immune cell populations, and measures of T-cell activation/dysfunction and correlated these to genetic features such as tumor mutation burden, neoantigen burden, mitochondrial mutations, and LOH from chromosomal uniparental disomy. Finally, immune profiles of patients with recurrence were compared with those of patients without recurrence.

HCC tumors exhibited low levels of immune infiltration, with the more aggressive widely invasive phenotype associated with more immune depletion. There was a negative correlation between tumor mutation burden, neoantigen burden, programmed cell death ligand 1 (PD-L1) expression, and the immune infiltration score. HCC tumors that exhibited a global LOH from chromosomal uniparental disomy or haploidization had the lowest level of immune infiltration. HCC tumors that recurred displayed an immune-depleted microenvironment associated with global LOH and aerobic glycolysis.

These findings offer new insights into the functional immune landscapes and immune microenvironment of HCC. Our data identify potential immunologic vulnerabilities for these understudied and often fatal cancers.

**Significance:**

The immune landscape of HCC is poorly defined and response rates to immunotherapy have not been reported. The authors found the immune microenvironment in HCC to be depleted. This immunosuppression is associated with a global LOH from haploidization and uniparental disomy, resulting in whole chromosome losses across the genome.

## Introduction

Hürthle cell carcinoma (HCC) is a rare type of cancer, representing 2% of all thyroid cancers. It is characterized by an abundance of dysfunctional mitochondria accounting for over 75% of the cell volume (1, 2). These cancers have a spectrum of behavior, ranging from the more indolent form (called minimally invasive HCC) to the aggressive form (called widely invasive HCC; refs. 3–6). Our group and others have reported the detailed genomic alterations that occur in these cancers, which show a high number of novel recurrent mutations, recurrent mitochondrial DNA (mtDNA) mutations, unique chromosomal alterations characterized by haploidization as well as whole chromosome duplication and uniparental disomy (7, 8). So far, there are little data on the immune features of HCC or the activity of immune checkpoint inhibitors in HCC. In recent years, drugs that inhibit T-cell checkpoints such as CTL-associated antigen 4 (CTLA-4) and programmed cell death protein 1/programmed cell death ligand 1 (PD-1/PD-L1) have shown remarkable efficacy in several cancers, including melanoma (9–12), non–small cell lung cancer (13–16), renal cancer (17–19), microsatellite instability-high carcinomas of various sites (20), and head and neck cancer (21, 22). Response to therapy has been correlated to critical molecular and immunologic features, such as tumor mutational load (9, 10, 13, 23, 24), human leukocyte antigens (HLA) genotype (25, 26), immune infiltration into the tumor microenvironment (27), CD8^+^ T-cell activation (10, 28), and expression of immune checkpoint molecules such as PD-L1 (28–30).

Our study aims to analyze the primary tumors of HCC to reveal the immunologic features of Hürthle cell tumors. This is critical for any potential development of immunologic interventions for these cancers.

## Materials and Methods

### Tumor Samples

Tumor and matched normal (peripheral blood or nonneoplastic normal tissue) specimens were obtained from 40 patients with HCC. Supplementary Figure S1 shows the demographic and clinical characteristics of the patients. All tissue samples were snap-frozen in liquid nitrogen at the time of surgery and stored at −80°C. Hematoxylin and eosin–stained tumor sections were reevaluated by a head and neck pathologist (R. Ghossein), confirming the diagnosis of HCC and the classification into either minimally invasive or widely invasive HCC. Our exact definition of these classifications follows: minimally invasive HCC (HMIN) was defined as encapsulated tumor harboring less than 4 foci of vascular invasion (foci of vascular invasion that were closely adjacent to one another were counted as separate foci) and lacking both gross and vascular invasions of extrathyroidal vessels. We defined widely invasive HCC (HWIDE) as a tumor with gross invasion/significant vascular invasion if the tumor was grossly invasive, had extrathyroidal vascular invasion, and/or was encapsulated with 4 or more foci of vascular invasion.

Written informed consent from patients for use of their tumor material was obtained, and the studies were approved by the Institutional Review Board at Memorial Sloan Kettering Cancer Center. All studies were conducted with recognized ethical guidelines according to the Belmont Report and Declaration of Helsinki.

### DNA and RNA Extraction

DNA was extracted using the DNeasy Blood & Tissue Kit (Qiagen) and quantified with the PicoGreen assay (Thermo Fisher Scientific). RNA was extracted using the RNeasy Mini kit (Qiagen) and quantified with the RiboGreen assay (Thermo Fisher Scientific). A Bioanalyzer (Agilent Technologies) and Fragment Analyzer (Advanced Analytics) were used to characterize DNA and RNA quality and integrity. We then prepared samples for whole-exome sequencing (WES) and RNA sequencing (RNA-seq).

### WES Libraries

We performed WES of the tumors and matched normal genomic DNA for 40 tumors to identify somatic mutations. WES libraries were prepared using the SureSelect XT library preparation kit (Agilent). DNA was sheared using a LE220 Focused-ultrasonicator (Covaris), and the fragments were end-repaired, adenylated, ligated to Illumina sequencing adapters, and amplified by PCR. We performed exome capture using the SureSelect XT v4 51Mb capture probe set (Agilent Bravo NGS, RRID:SCR_019473) and captured exome libraries were enriched by PCR. Final libraries were quantified using the KAPA library quantification kit (KAPA Biosystems), Qubit fluorometer (Life Technologies), and 2100 Bioanalyzer (Agilent), and were sequenced on a HiSeq 2500 sequencer (Illumina) using 2×125-bp cycles with a depth of coverage of > 100×.

### RNA-seq Libraries

We used the KAPA Stranded RNA-seq with RiboErase sample preparation kit (KAPA Biosystems) to prepare RNA-seq libraries. Total RNA (100 ng) was ribo-depleted and fragmented, followed by first- and second-strand synthesis, A tailing, adapter ligation, and PCR (using 11 cycles). We quantified the final libraries using the KAPA library quantification kit, Qubit fluorometer, and 2100 Bioanalyzer, and then sequenced them on a HiSeq 2500 v4 chemistry sequencer using 2×125-bp cycles with a depth of coverage of > 100×.

### Mutation Analysis

We confirmed matches between tumor and normal samples for each patient with fingerprinting analysis using an in-house panel of 118 SNPs and with VerifyBamID (31). Raw sequencing data were aligned to the hg37 genome build using the Burrows-Wheeler Aligner (RRID:SCR_010910) version 0.7.17 (http://www.ncbi.nlm.nih.gov/pmc/articles/PMC2705234/). Further indel realignment, base-quality score recalibration, and duplicate-read removal were performed using the Genome Analysis Toolkit (GATK, RRID:SCR_001876) version 3.8 (http://www.ncbi.nlm.nih.gov/pmc/articles/PMC2928508/) following raw reads alignments guidelines (http://www.nature.com/ng/journal/v43/n5/pdf/ng.806.pdf).

Single-nucleotide variant (SNV) and short insertions/deletions (INDELS) callers used were VarScan v2.4.3 (32), Strelka v2.9.10 (33), Platypus 0.8.1 (34), Mutect2—part of GATK 4.1.4.1 (35), Somatic Sniper version 1.0.5.0 (36). SNVs were identified by at least two different callers. SnpEffect and SnpSift version 4.3 programs were used for annotating and predicting the effects of SNPs (37). Variants were called for Tcov > 10 and Taf ≥ 0.04 and Ncov >7 and Naf ≤ 0.01 and Tac >4. Common SNPs are eliminated by comparison with snp142.vcf. Rare variants found in the Single Nucleotide Polymorphism Database were kept if Naf = 0. Variants with Tcov <20 or Tac <4 were marked as low_confidence. We applied additional optimization and filtering for INDELS. INDELS in blacklisted regions (https://www.encodeproject.org/annotations/ENCSR636HFF/) and low mappability regions (such as repeat maskers) were excluded.

#### Telomerase Reverse Transcriptase Mutation Testing

Telomerase reverse transcriptase (*TERT*) promoter mutations were identified from the sequencing data of tumors. The *TERT* proximal promoter was also amplified from genomic DNA as described previously (7).

### Somatic Mitochondrial Mutations

Aligned reads were analyzed using a custom informatics pipeline for mtDNA analysis as described previously (7).

### Copy-number Analysis by FACETS and FISH

To characterize allele-specific somatic DNA copy-number alterations, we applied FACETS to the tumor and normal tissue pairs of BAM files (38). Copy-number alterations, tumor purity, ploidy, and cellular fractions were estimated and reported as described previously (7). FISH analysis was performed on formalin-fixed, paraffin-embedded sections using a 3-color probe designed to confirm the copy-number changes of chromosomes 2, 5, and 7 detected by FACETS (7). We categorized tumors according to three types: diploid, near-haploid, and polysomic. Diploid tumors had two copies of each chromosome, with or without focal alterations in individual chromosomes. Near-haploid tumors had single copies of the majority of chromosomes, except for chromosome 7 (which was always diploid) and chromosome 5 (which was diploid in some cases). Polysomic tumors had whole chromosome gains of chromosome 7 (usually whole chromosome duplication, resulting in four copies), as well as, in some cases, chromosome 5 and chromosome 12. Tumors that were either haploid or polysomic from uniparental disomy were categorized as having global LOH.

### Gene Expression Analyses of Tumors

RNA-seq raw read sequences were aligned against human genome assembly hg19 by STAR two-pass alignment. Gene-level count values were computed by the summarizeOverlaps function from the R package “GenomicAlignments” with hg19 KnownGene as the base gene model. The Union counting mode was used and only mapped paired reads were considered. Fragments per kilobase of exon model per million reads mapped (FPKM) values were then computed from gene-level counts by using the FPKM function from the R package “DESeq2” (DESeq, RRID:SCR_000154). After principal component analysis to visualize possible batch effects, the ComBat batch correction method was used (39) through implementation in the R sva package (40). Several orthogonal tools for the deconvolution of immune infiltration from RNA-seq data were implemented as indicated below.

#### Immune Infiltration and Immune Activity Analyses

To assess immune infiltration and activity in tumors using bulk RNA-seq data, we applied several orthogonal tools. Cell-type identification by estimating relative subsets of RNA transcripts (CIBERSORT) employs a reference gene expression signature and performs a linear support vector regression to adaptively select genes from the reference (41). Single-sample gene set enrichment analysis (ssGSEA; ref. 42) calculates enrichment scores for a sample and gene set pair, allowing clustering by pathways rather than individual genes, and generates metrics such as immune infiltration score (IIS) and T-cell infiltration score (TIS) as described by Şenbabaoğlu and colleagues (43) IIS is an aggregate score for innate and adaptive immune scores, while TIS is an aggregate score of 9 T-cell subtypes. Estimation of stromal and immune cells (IC) in malignant tumor tissues using expression data (ESTIMATE) is an ssGSEA-based technique, which uses differential gene expression from high and low IC infiltrating tumor samples to derive a 141-gene signature estimating the degree of stromal and immune infiltration in a tumor (bioinformatics.mdanderson.org/estimate; ref. 44). Immune cytolytic activity (“CYT” score) is calculated from geometric means of transcript levels of the two effector genes: granzyme A (GZMA) and perforin (PRF1; ref. 45). Batch-corrected normalized data were used for input into the Tumor Immune Dysfunction and Exclusion model (46).

#### Differentially Expressed Gene and Pathway Analysis

Five tumor samples with the highest and the lowest IIS per HCC histology were used for differentially expressed gene analysis, which was performed through the DESeq2 package in R. Using the raw count data and gene model, DESeq2 normalized the expression raw count data by sample-specific size factor while testing for genes found with a significantly different expression between the high IIS group and the low IIS group samples. Canonical pathway analysis of differentially expressed genes was performed using Qiagen Ingenuity Pathway Analysis (Ingenuity Pathway Analysis, RRID:SCR_008653).

#### Comparison of Immune Scores with Other Cancers

We used publicly available RNA-seq data from The Cancer Genome Atlas (TCGA, https://portal.gdc.cancer.gov/) dataset cohorts to compare the tumor immune microenvironment with the HCC samples: these included bladder urothelial carcinoma (*n* = 95), colorectal adenocarcinoma (*n* = 336), breast cancer (*n* = 1,205), head and neck squamous cell carcinoma (*n* = 293), kidney renal clear cell carcinoma (*n* = 658), glioblastoma multiforme (*n* = 123), lung adenocarcinoma (*n* = 228), ovarian serous cystadenocarcinoma (*n* = 248), lung squamous cell carcinoma (*n* = 258), and uterine corpus endometrial carcinoma (*n* = 534).

### IHC

Formalin-fixed paraffin-embedded tissue sections of tumors were sectioned onto glass slides at 4 μm thickness. Consecutive tissue sections were stained with antibodies by the Molecular Cytology Core Facility at Memorial Sloan Kettering Cancer Center using the Discovery XT processor (Ventana Medical Systems). Hematoxylin and eosin stains were performed under standard procedures and reviewed by two head and neck pathologists (B. Xu and R. Ghossein) to confirm the histologic diagnosis and evaluate additional immunostaining. Serial unstained slides (4 μm) were prepared from each block for subsequent IHC with the following antibody clones: PD-L1 (clone:E1L3N, dilution: 1:400, Cell Signaling Technology), PD-1 (clone: NAT105, ready to use RTU, Cell Marque), CD4 (clone: SP35, dilution 1:12.5, Cell Maque), CD8 (clone: SP57, RTU, Ventana Medical Systems), FOX P3 (236A/E7; Abcam), Granzyme B (Ventana Medical Systems, catalog no. 760-4283). The sections were stained on the Ventana Benchmark Ultra automated staining platform (Ventana Medical Systems) or on Leica Bond-III Autostainer (Leica Biosystems), according to the manufacturer's instructions. Each IHC stain was evaluated and qualified by a head and neck pathologist (B. Xu). For PD-L1, positive tumor cell (TC) staining was defined as either partial or complete membranous staining of any intensity. Positive IC staining was defined as cytoplasmic or membranous staining of any intensity. Only tumor-infiltrating ICs were included in IC scoring. The combined positive score was defined as the number of PD-L1–positive TCs and ICs divided by the total number of TCs × 100. For all other IHCs, the number of ICs positive for each stain was counted manually at 400X magnification (field diameter 0.55 mm) at the hotspot. Hotspot is defined as a high-power field with the highest density of positive ICs.

### Mutation-associated Neoantigen Identification and HLA Affinity Prediction

Mutation-associated neoepitopes were identified and binding to HLA was predicted by NetMHCpan 4.0 (47), with patient-specific HLA type determined using PolySolver (48). HLA zygosity and allele-specific copy-number analyses were performed using FACETS (38).

### Statistical Analyses

We performed the D'Agostino–Pearson omnibus normality test to determine whether datasets follow a Gaussian distribution in each comparison. If the data were Gaussian, parametric tests were performed (two-tailed unpaired *t* tests, one-way ANOVA with Tukey correction for multiple comparisons, or Pearson correlation). If the data were non-Gaussian, nonparametric tests were applied (Mann–Whitney *U* test, one-way ANOVA using Kruskal–Wallis with Dunn correction for multiple comparisons, or Spearman correlation). The *a priori* definition of statistical significance for all hypothesis testing was two-tailed α < 0.05. An outcomes analysis was performed using the Kaplan–Meier method and compared using the log-rank test.

### Data Availability

The data generated in this study, beyond the information provided in the main text and supplementary figures, are available upon request from the corresponding authors. RNA-seq data used for the immune deconvolution analysis have been deposited in sequence read archive (SRA) with reference number PRJNA989587. The data will be available at https://www.ncbi.nlm.gov/sra/PRJNA989587.

## Results

### Patient, Tumor, and Treatment Characteristics

A cohort of 40 patients undergoing treatment for HCC [21 widely invasive (HWIDE) and 19 minimally invasive (HMIN)] at Memorial Sloan Kettering Cancer Center was analyzed for this study. This represented a subset of the 56 patients with HCC, about whom we have previously published findings (7). Median follow-up time was 45 months. Clinicopathologic characteristics (including age, sex, stage, and pathologic subtype), treatment characteristics (surgery and use of radioactive iodine), and outcomes (locoregional recurrence, distant recurrence, and survival) are described in Supplementary Fig. S1. Compared with patients with HMIN HCC, patients with HWIDE HCC had larger tumors (T3/T4) with extrathyroidal extension (*P* = 0.016), presented more frequently with stage III or IV disease (*P* = 0.002), and were more likely to have locoregional (*P* = 0.018) and distant (*P* = 0.034) metastatic recurrences.

### Immune Infiltration and the Tumor Microenvironment of HCC

#### Immune Infiltration of HCC Compared with Other Tumor Types and by HCC Phenotype

**FIGURE 1:**
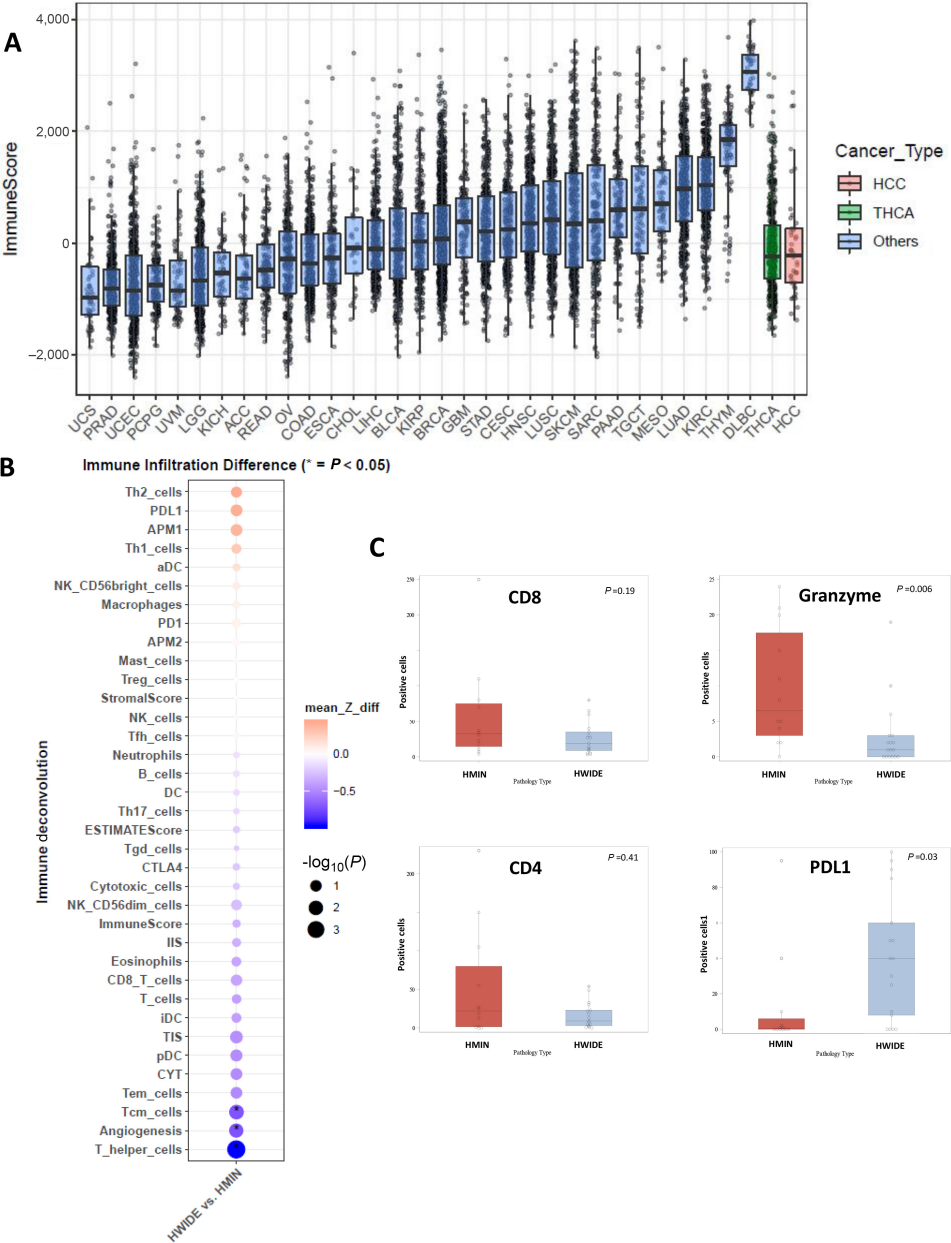
The immune landscape of HCC compared with TCGA tumors is shown in **A**. Immune infiltration differences between HWIDE tumors with HMIN tumors are shown in **B** using RNA-seq data and in **C** using IHC.

Tumors underwent RNA-seq and normalized data were analyzed to quantify and deconvolve immune infiltration using several methods, including CIBERSORT (41), ESTIMATE (44), ssGSEA (43), and cytolytic (CYT) score (45). We then compared the immune infiltration observed in HCCs with other tumor types from TCGA. We also directly compared the immune infiltration of HCC with papillary thyroid cancer. We found that HCC has a relatively immune-depleted microenvironment (Fig. 1A) comparable with that of papillary thyroid cancer (PTC). When we stratified by HCC type, we observed that the more aggressive HWIDE tumors have fewer Th cells and central memory T cells and a lower CYT score compared with the less aggressive HMIN tumors (Fig. 1B). In cases with sufficient tissue available, we also carried out IHC analysis on select markers (Fig. 1C). There was a significant decrease in activated T cells (indicated by granzyme B staining, *P* = 0.006) in the HWIDE tumors. CD4^+^ cells and CD8^+^ cell counts were also lower, but this was not significant due to the low sample size (Supplementary Table S1). We also examined the expression of checkpoint inhibitor molecule PD-L1 stratified by HCC phenotype. This showed increased expression in the HWIDE phenotype (*P* = 0.03).

### Deconvolution of Immune Infiltration and Relationship to Tumor Mutation and Neoantigen Burden

**FIGURE 2:**
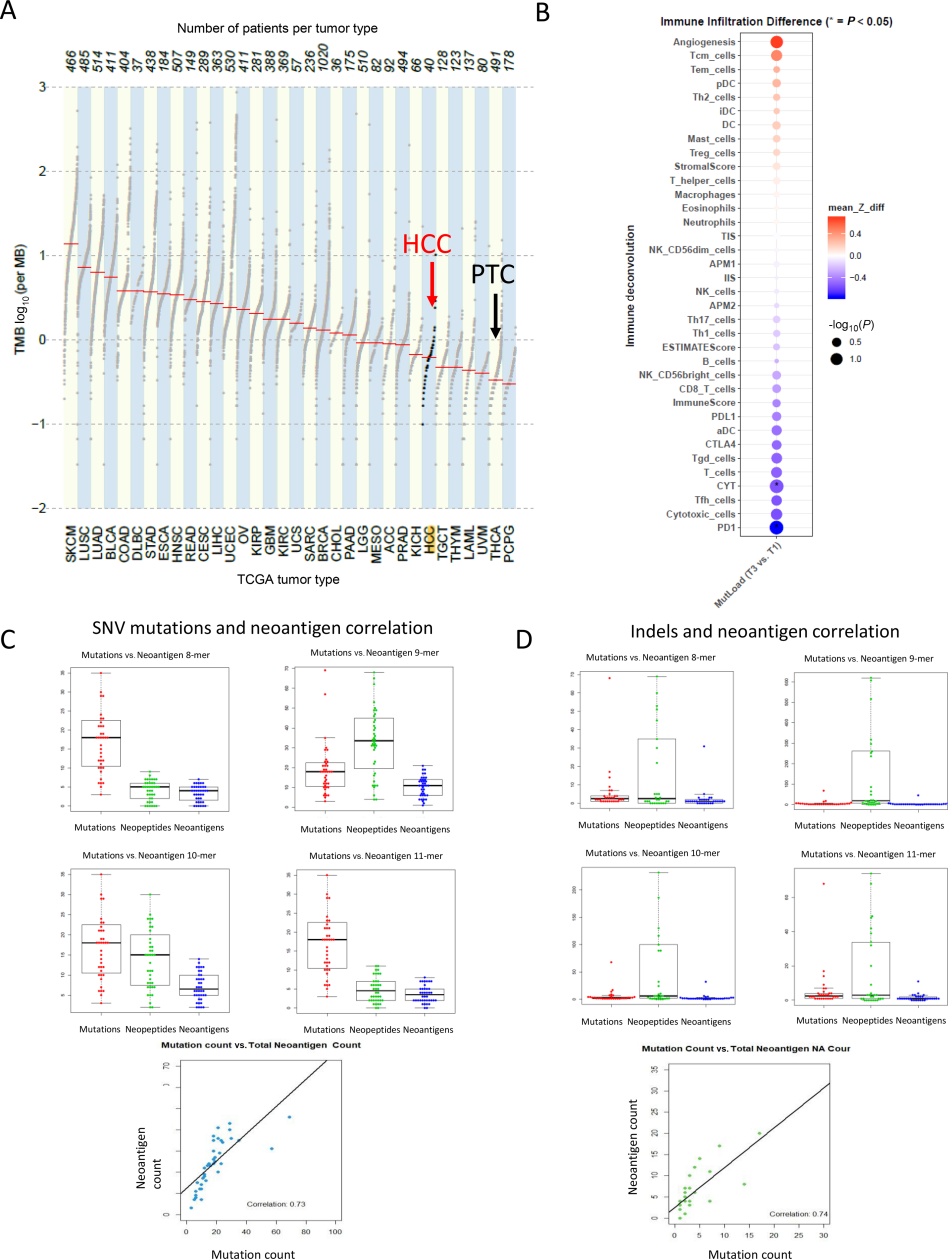
The TMB for HCC was compared with other tumors in TCGA (**A**). The association between TMB and immune infiltration is shown in **B**. Correlation of the number of neoantigens generated by SNVs (SNV-NeoAg) with the number of SNVs in the tumor is shown in **C**. Correlation of the number of neoantigens generated by INDELS with the number of SNVs in the tumor is shown in **D**. Correlation of tumor mutation load, neoantigen load, and tumor infiltration score is shown in Supplementary Fig. S3.

We wanted to analyze the relationship between tumor mutation burden (TMB), neoantigen burden, and immune infiltration. We have previously reported the exome sequencing on 56 HCC tumors (7). Of these 56 tumors, deconvolution data of immune infiltration were possible in 40 tumors. Therefore, our analysis of the association between mutation burden and neoantigen burden was based on these 40 tumors. The detailed mutation profile of these 40 tumors is shown in Supplementary Table S2 and Supplementary Fig. S2. In these 40 tumors, 1,136 somatic mutations and indels were identified with the widely invasive phenotype having significantly more mutations than the minimally invasive phenotype (mean 40 ± 62 vs. 15 ± 7; *P* = 0.04). As we have reported previously (7), the most frequently mutated genes included *MADCAM1* (12%), *EIF1AX* (10%), *NF1* (10%), *PTPRS* (10%), *NRAS* (8%), and *TP53* (8%; Supplementary Fig. S2A). The majority of mutations were SNVs with a small percentage of deletions and insertions (Supplementary Fig. S2B). Missense mutations were most common followed by splice site mutations, frameshift deletions, and nonsense mutations. The most common DNA substitution types were C>T transitions, followed by T>C transitions, C>A transversions, C>G transversions. The distribution of DNA substitution type per tumor is shown in Supplementary Fig. S2C. Comparing the TMB with other types of cancer in TCGA (TCGA abbreviations are listed at https://gdc.cancer.gov/resources-tcga-users/tcga-code-tables/tcga-study-abbreviations), the TMB of HCC was low, comparable with kidney chromophobe cancer and testicular germ cell tumors (Fig. 2A). However, the TMB was greater than that observed for papillary thyroid cancer. We next analyzed the association between TMB and immune infiltration (Fig. 2B; Supplementary Fig. S3). Tumors with the highest TMB (tumors in the upper quartile of the cohort labeled as T3) were compared with tumors with the lowest TMB (indicated by T1 in the lowest quartile). In general, tumors with high tumor mutation load respond well to checkpoint inhibitor immunotherapy. However, high TMB does not necessarily correlate with pretreatment immune infiltration. Interestingly, in HCC, we observed that tumors with higher mutation load (T3) tended to have low immune infiltration, which included lower Th cells, cytotoxic T cells as well as lower expression of PD-L1 and PD-1.

The mutation-derived neoantigens predicted to bind to patient-specific HLA class I were then identified using netMHCpan. SNV- and INDEL-associated neoantigen counts are summarized per sample in Supplementary Tables S3–S10. As expected, the number of predicted neoantigens generated by SNVs (SNV-NeoAg) was tightly correlated with the number of SNVs in the tumor (Pearson *r* = 0.73, *P* < 0.0001; Fig. 2C). The number of neoantigens generated by INDELS (INDEL-NeoAg) was lower than SNV-Neoag counts but was again tightly correlated with the number of INDELs (Pearson *r* = 0.74, *P* = 0.001; Fig. 2D). We next analyzed associations between SNV load, SNVneoantigen load, and the TIS. Again, we found a negative correlation between SNV load, neoantigen load, and TIS (*r* = −0.34, *P* = 0.04; Supplementary Fig. S3).

We next went on to determine the immune infiltration microenvironment of HCC and whether this had any correlation to common genomic alterations in HCC such as mitochondrial mutations, TERT mutations, and the HCC chromosomal landscape.

### Tumor Immune Infiltration Stratified by Mitochondrial Mutation, TERT Mutation, and Major LOH

**FIGURE 3:**
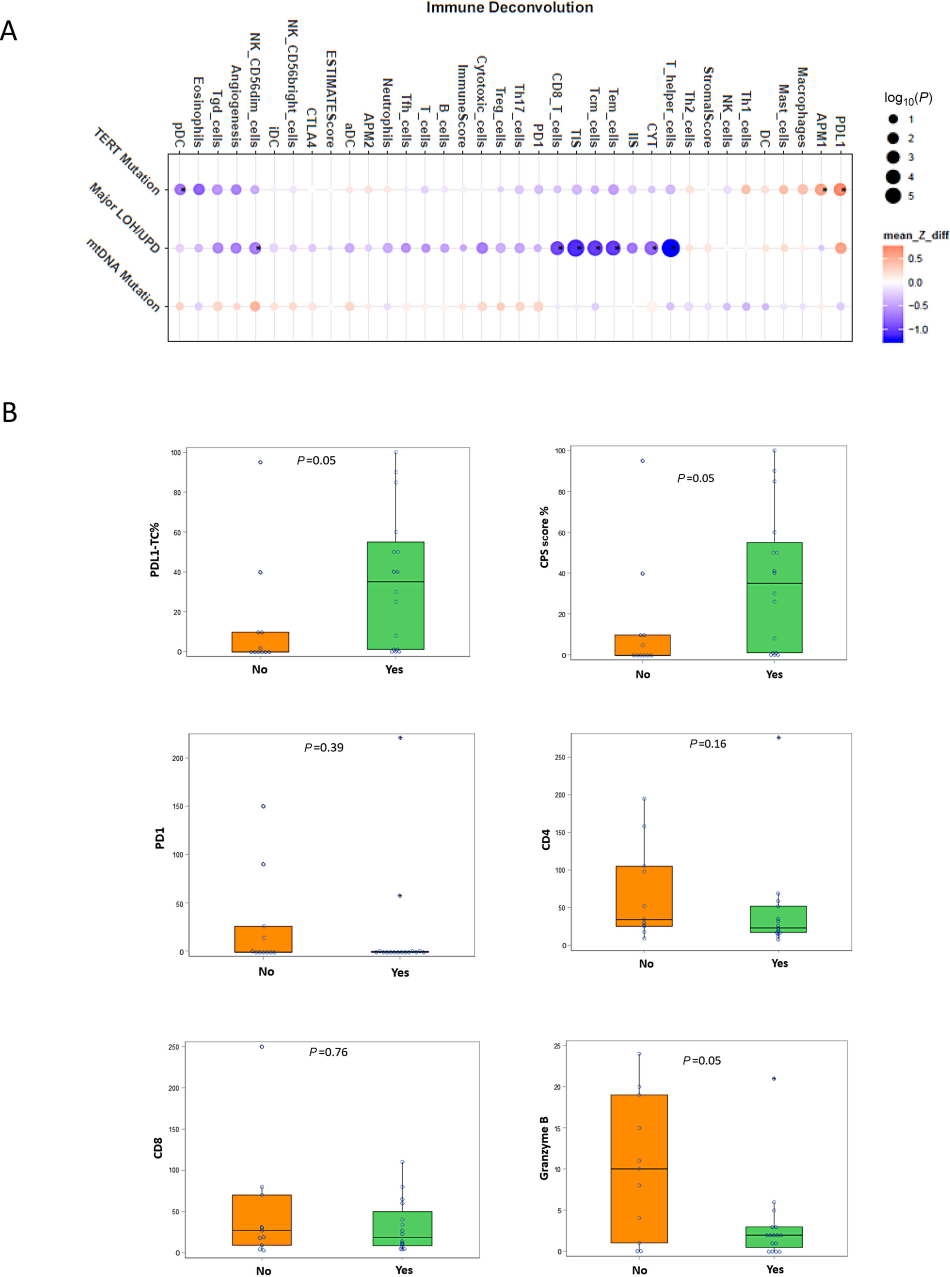
The immune landscape of HCC stratified by the presence of mitochondrial mutations, TERT promoter mutations, and global LOH is shown in **A** using RNA-seq data. Tumors with global LOH have very low TIS, low CD8 cytotoxic T cells, CD4 helper cells. The immune landscape of tumors with global LOH compared with those without global LOH by IHC is shown in **B**. The box plots showing each individual change by RNA-seq are illustrated in Supplementary Fig. S4. Tumors with global LOH have very low TIS, low CD8 cytotoxic T cells, CD4 helper cells, central memory T cells, effector memory T cells. Validation by IHC is shown in B.

Our previous studies have shown that HCC tumors are characterized by recurrent mitochondrial mutations, high TERT promoter mutations, and unique chromosomal alterations, with some tumors showing major LOH from haploidization or uniparental disomy (7). We, therefore, wanted to examine the immune infiltration of HCC stratified by these different molecular features, which is shown in Fig. 3A. A characteristic of HCC is the presence of a high number of mitochondrial mutations, particularly those in complex I of the electron transport chain, which results in dysregulation of the tricarboxylic acid cycle. When we stratify the immune profile by the presence of these mtDNA mutations, we see no major differences, suggesting that mitochondrial mutations may not impart any specific alterations to the immune microenvironment. For tumors with TERT mutations, there is increased expression of PD-L1 and also M1 antigen-presenting macrophages. Finally, we correlated the chromosomal landscape to the immune profile. We have previously reported that HCC shows unique chromosomal landscapes with haploidization and uniparental disomy being characteristic features, especially in the widely invasive phenotype (7). The effect of these chromosomal abnormalities is a massive global LOH. When we stratify the immune infiltration landscape by this LOH, we see quite striking features. Tumors with major LOH have statistically significant lower TIS. This includes lower CTLs (*P* = 0.013), CD8 cells (*P* = 0.005), CD4 helper cells (0.00008), central memory T cells (*P* = 0.0003), effector memory T cells (0.003), regulatory T cells (*P* = 0.032). These LOH tumors also show low CYT scores (*P* = 0.0019). The box plots demonstrating each change are shown in Supplementary Fig. S4. The corresponding IHC scores stratified by LOH are shown in Fig. 3B. These findings suggest that the global LOH as a result of haploidization and uniparental disomy results in a striking immune-depleted microenvironment, which would favor poorer response rates to immunotherapy. Expression of checkpoint inhibitor molecules PD-L1 and PD-1 stratified by LOH was also determined by mRNA expression; protein expression was determined by IHC. Interestingly, tumors with LOH had increased expression of PD-L1 (*P* = 0.06) and combined positive scores (*P* = 0.06).

### Genes Mediating Decreased Immune Infiltration in Tumors with Global LOH

In HCC, the LOH observed is a global LOH due to loss of whole arm chromosomes rather than focal losses with the exception of specific chromosomes 5,7,12. We carried out differential gene expression analysis comparing tumors with major LOH to tumors without major LOH to identify whether there were immunoregulatory pathways and genes associated with immune infiltration that were decreased. The regulation of immune infiltration is a complex process involving multiple genes and pathways. There are many genes which can influence immune infiltration. These include chemokines which are small signaling proteins that attract cells to specific locations. Chemokines such as CCL2, CCL3, CCL5, and CXCL9 can promote immune cell infiltration by attracting monocytes, macrophages, T cells. Cytokines can also increase immune infiltration. These are signaling molecules that regulate immune responses. Genes encoding cytokines include the ILs, IFNs, and TNF. Adhesion molecules are also important. These molecules mediate the attachment of immune cells to the endothelial cells lining blood vessels facilitating their infiltration into tissues. Genes encoding adhesion molecules like selectins, integrins, and immunoglobulin superfamily such as ICAM1 and VCAM1 are involved in immune cell infiltration. Toll-like receptors (TLR) are a class of pattern recognition receptors that recognize specific components of pathogens and activate immune responses. Activation of TLR signaling can induce the production of chemokines and cytokines promoting immune cell infiltration. Various TLR genes, such as TLR4, TLR7, and TLR 9 are associated with immune infiltration. MHC genes are important. MHC molecules play a crucial role in presenting antigens to immune cells, triggering immune responses. Genes within the MHC region include the HLA genes among others.

**FIGURE 4:**
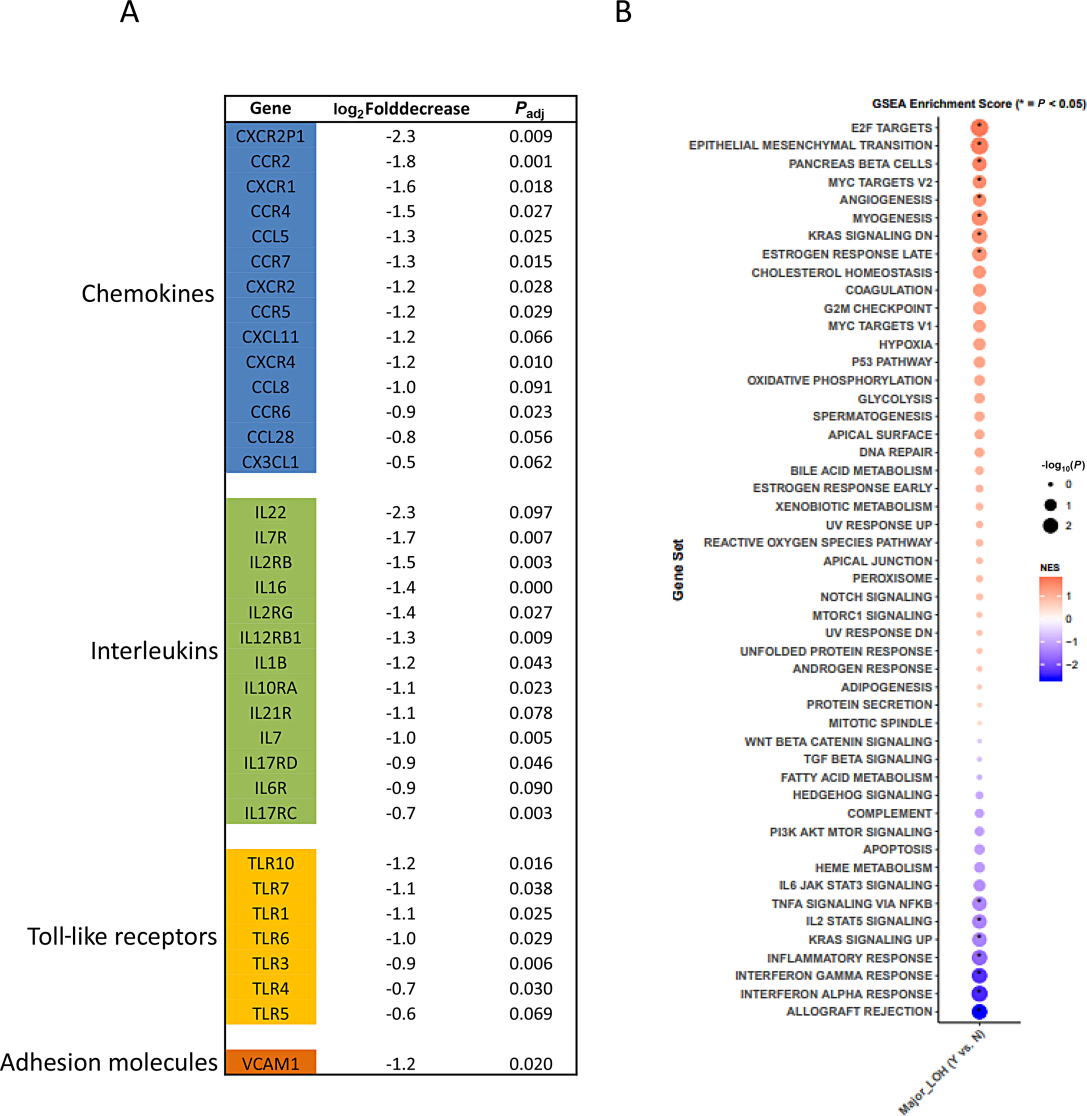
Differential gene expression of immune infiltration genes of tumors with global LOH compared with tumors without global LOH is shown in **A**. Gene set enrichment analysis showing pathways significantly altered is shown in **B**.

We have analyzed the DEG of all of these different genes and this is shown in Fig. 4. We see significant decrease in expression of 14 different chemokine genes, 13 different ILs, seven different TLRs, and one adhesion gene. Gene set enrichment analysis of hallmark gene pathways show significant decrease in IFN alpha response, IFN gamma response, inflammatory response, allograft rejection, IL2 STAT5 signaling, TNFA signaling. Therefore, overall this confirms the decrease in immune infiltration is mediated through a variety of different pathways and genes.

### Immune Infiltration Correlates with Recurrence

**FIGURE 5:**
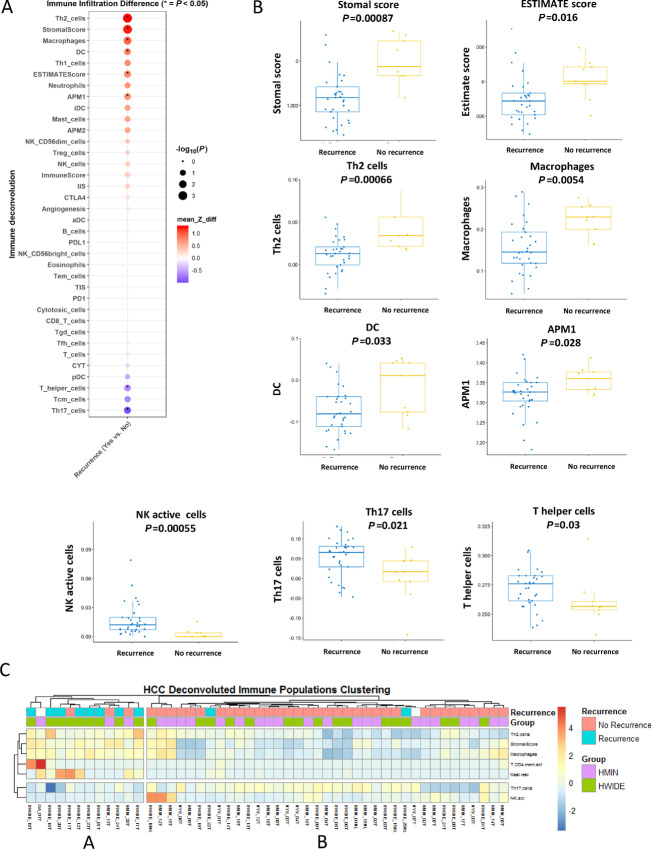
The immune landscape of HCC using ssGSEA stratified by recurrence is shown in **A**. Corresponding box plots are shown in **B**. Unsupervised clustering of the immune gene expression profiles of these cell differences showed two cohorts of tumors, group A and B, with group A being enriched with the tumors that recurred (**C**).

To determine whether the level of T-cell infiltration in the tumor microenvironment was associated with clinical outcome, we compared HCCs that developed recurrence (either locoregional or distant), with those that did not recur (Fig. 5A). Patients with recurrence had tumors with higher Th2 cells, stromal scores, macrophages, dendritic cells, APM1 cells, and a higher ESTIMATE score (Fig. 5B). They also had lower natural killer (NK) cells, Th cells, and Thy17 cells. Unsupervised clustering of the immune gene expression profiles of these cell differences showed two cohorts of tumors, groups A and B, with group A being enriched with the tumors that recurred (Fig. 5C). These data are consistent with a relative lack of immune surveillance that may be associated with the ability of HWIDE to develop recurrence or distant metastasis.

### HLA Genotype Diversity in HCC Tumors

We then examined whether the tumors that recur utilize loss of HLA, which would impair the ability to present a diverse neoantigen repertoire, as a means of immune evasion. Using FACETS (38) to call allele-specific copy number at the HLA class I locus in each case with exome sequencing of normal DNA, we determined which cases harbored either single-copy loss of HLA (hemizygous or copy-neutral LOH) or at least one homozygous HLA class I allele (26). We calculated the HLA divergence score (26) for HLA A, HLA B, and HLA C as well as the mean overall HLA divergence (Supplementary Tables S11 and S12). This analysis revealed that there was no difference in the mean HLA divergence score nor in the individual HLA types in patients who recurred and in patients who did not recur. This suggests that more aggressive HWIDE tumors that recur do not employ HLA genetic loss as an immune escape mechanism.

### Canonical Pathways Involved in Recurrence

**FIGURE 6:**
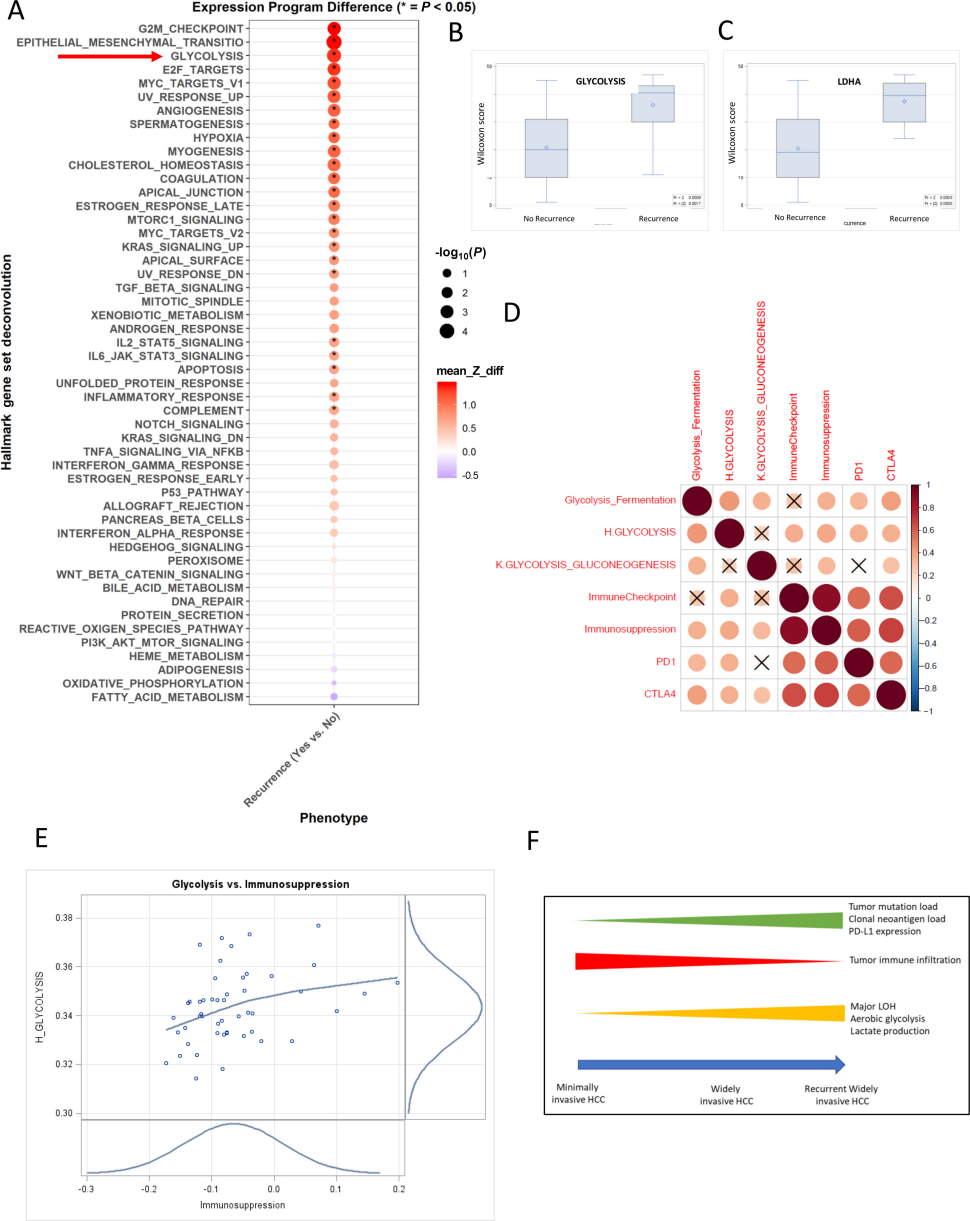
Pathways dysregulated by immune landscape genes in recurrent HCC. Pathways significantly enriched in patients with recurrence included epithelial–mesenchymal transition, glycolysis, angiogenesis, hypoxia, mTOR signaling, MYC signaling (**A**). When we compare the mRNA expression of genes involved in glycolysis (**B** and **C**), tumors that recur have significant overexpression of these genes. Spearman correlation matrix plot (**D**) showing positive correlation between glycolysis signatures (Glycolysis/fermentation, Hallmark Glycolysis, KEGG glycolysis-gluconeogenesis) and immune suppression signatures (Immune Checkpoint, immunosuppression, PD-1, CTLA-4) with “X” indicating nonsignificant correlation. Scatter plot of glycolysis versus immunosuppression (**E**) with density and loess regression (Spearman correlation 0.39, *P* = 0.006). A summary of the alterations that impact immune response correlated to HCC phenotype is shown in **F**.

We subsequently examined the transcriptional pathways that were differentially activated between tumors that recurred and those that did not (Fig. 6). The transcriptomes from samples with recurrence were compared with those that did not recur. We used Ingenuity Pathway Analysis (Qiagen) of significantly differentially expressed genes to identify enriched canonical pathways. Among the pathways that were significantly enriched in patients with recurrence included epithelial–mesenchymal transition, glycolysis, angiogenesis, hypoxia, mammalian target of rapamycin signaling, and MYC signaling (Fig. 6A). The observation of glycolysis being activated in the tumors with recurrence is compatible with the metabolic profile of HCC tumors that show a switch to aerobic glycolysis from dysregulation of the electron transport chain from mtDNA mutations affecting complex I of the electron transport chain. When we compared the mRNA expression of the lactate dehydrogenase gene (Fig. 6B) and genes involved in glycolysis (Fig. 6C) we saw that those tumors that recurred had significantly elevated expression of these genes (*P* = 0.0005 and *P* = 0.017, respectively). We then correlated glycolysis signatures, like Glycolysis/Fermentation, Hallmark Glycolysis, and Kyoto Encyclopedia of Genes and Genomes (KEGG) gluconeogenesis with immune suppression signatures, like ImmuneCheckpoint, Immunosuppression, PD-1, and CTLA-4. In Fig. 6D and E, we show statistically significant associations between the glycolysis signatures and immunosuppression signatures. This may suggest that the increased expression of lactate, generated from aerobic glycolysis, is potentially associated with the immune poor environment seen in patients with recurrence. Figure 6F summarizes the alterations that impact immune response correlated to HCC phenotype.

## Discussion

HCC is a rare subtype of thyroid cancer accounting for 2% of all well-differentiated thyroid cancer. Of these, 30% comprise the more aggressive subtypes with extensive angioinvasion. These aggressive subtypes do not express thyroid differentiation genes and therefore the use of radioactive iodine is ineffective (7). Overall, approximately 15% of patients will die of disease due to the development of distant metastatic disease. At present, there is no effective chemotherapy agent for these patients. Therefore, there remains a desperate need for new therapies to be developed to treat these patients. Understanding the immune landscape of these cancers may reveal possible immunotherapy approaches to treat these patients.

Our study aim was to analyze the primary tumors of HCC to describe the immunologic phenotypes of this cancer. To do this, we systematically examined the molecular features that are reported to be associated with tumor immunity or response to immunotherapy. This includes features such as tumor mutational load (9, 10, 13, 23), clonal neoantigen load (24), HLA genotype (25, 26), immune infiltration into the tumor microenvironment (27), and expression of immune checkpoint molecules such as PD-1 and PD-L1 (28–30). In our study, we report that HCC has a relatively low TMB compared with other tumors in TCGA. However, the more aggressive forms of HCC do have both a higher mutational load and a higher clonal neoantigen load as well as higher expression of PD-L1–expressing TCs compared with the more indolent forms of HCC. This could suggest that the more aggressive cancers may be amenable to checkpoint inhibitor drugs, particularly drugs that target the PD-L1 ligand expressed on TCs and antigen-presenting cells. One of the most striking findings of our study was the observation that these cancers have a relatively low immune infiltration, which was most marked in the more aggressive, widely invasive phenotype of HCC. Moreover, tumors that recur displayed the same depletion of ICs in addition to a higher stromal score, as well as higher macrophage and dendritic cell infiltration. TMB reflects the frequency of somatic mutations that give rise to neoantigens. Neoantigens increase the tumor immunogenicity by activating CD8^+^ CTLs that initiate TC lysis. Immune checkpoint response depends on reinvigorating the CYT potential of CTLs to eliminate TCs. It is unknown what type of response to immunotherapy HCC tumors would have. Reports by Cristescu and colleagues (49) divided tumors into TMB high/low and T-cell infiltration high/low and showed that tumors with a mixed picture (e.g., TMB high/T-cell low or TMB low/T-cell high) may respond.

We identified some novel findings that could explain why we see an immune-depleted microenvironment in HCC. First, we have previously described that HCC has very unique major chromosomal alterations displaying chromosomal duplication of chromosomes 5, 7, and 12 coupled with uniparental disomy of the remaining chromosomes (7). This widespread uniparental disomy results in a global LOH in these cancers. We report a very striking correlation between this major LOH and the depletion of immune infiltration in these tumors, particularly in Th cells, CTLs, and NK cells. This lower immune infiltration was mediated through multiple genes and pathways with a decrease in expression of genes coding for chemokines, ILs, TLRs, and adhesion molecules. Thus, even though the aggressive forms of HCC have a higher mutational and neoantigen load, it appears that the global LOH resulting from chromosomal alterations is linked to immune infiltration depletion. It is unclear what the mechanism is to explain this association. A study by Davoli and colleagues (50) explored the association of somatic copy-number alterations (SCNA) with immune evasion. In this study, SCNAs were categorized into groups by high and low number of SCNAs and then correlated with gene expression profiles of cell signaling pathways and with markers for cytotoxic IC infiltrates (immune infiltration). They found that tumors with high levels of arm and whole chromosome SCNAs had low expression levels of immune signature and that SCNA was a stronger predictor of immune infiltration than TMB. They also analyzed the responses to immunotherapy in two clinical trials on patients with melanoma and found that patients with high levels of SCNA had poorer outcomes. The authors hypothesized that protein imbalance may impair a tumor signal needed for cytotoxic IC infiltration. The arm and whole chromosome alterations may weaken some aspects of antigen presentation on the MHC. An alternative hypothesis may be related to the relative concentration of neoantigen peptides in high versus low aneuploidy tumors. The relative concentration of the average neoantigen in a triploid tumor would be 33% lower than the same antigen in a diploid tumor. It is also possible that the increased flux of unstable wild-type proteins through the proteasome generates more self-peptides placing neoantigens at a further disadvantage. It is therefore possible that the unique whole chromosome alterations prevalent in HCC may contribute to the suppressed immune activities we observed. We therefore carried out differential gene expression analysis comparing tumors with global LOH to tumors without global LOH to determine whether tumors with high global LOH had low levels of genes involved in immune infiltration. We indeed found a decrease in expression of genes coding for 14 different chemokines, 13 ILs, seven TLRs, and one adhesion molecule. Pathways involved in immune function were also decreased.

Another potentially significant finding was the metabolic impact that these tumors could have on the immune system. HCC is characterized by mitochondrial mutations that result in the disengagement of the electron transport chain from the citric acid cycle resulting in a switch toward aerobic glycolysis as a means of energy production (51). Recently, we reported on the metabolic landscape of HCC and showed HCC tumors have a profound depletion of citrate pools with impairment of the tricarboxylic acid cycle and switch to aerobic glycolysis (51). We also identified that metabolites in the reduced form of nicotinamide adenine dinucleotide–dependent lysine degradation pathway were elevated exclusively in HCC and showed an increase in unsaturated fatty acid metabolism with an accumulation of polyunsaturated fatty acids. The more aggressive forms of HCC that recur display more pronounced aerobic glycolysis, resulting in high levels of lactate being produced. Recently, several studies reported that high lactate levels have a direct impact on many aspects of the immune system with inhibition of Th cells, CTLs, NK cells, and dendritic cells (52–54). High lactate also promotes the conversion of macrophages from the M1 phenotype to the M2 immunosuppressive phenotype (54). Thus, the higher lactate environment associated with the more aggressive forms of HCC may also be responsible for the immune-depleted microenvironment we observed and may also impair function of any T cells present within the tumor. Indeed, our analysis showed an association between the glycolytic signatures of tumors with immune suppression signatures, which is in agreement with these other reports.

Whether immune checkpoint inhibitor drugs will show efficacy in HCC is still to be determined. Clinical trials are currently planned either alone or in combination with drugs such as lenvatinib (55) that show efficacy in aggressive forms of thyroid cancer. The response will depend upon the balance between favorable and unfavorable factors. The presence of a higher mutation load, neoantigen load, and PD-L1 expression is favorable for response to therapy. However, the low immune infiltration associated with global LOH and lactate production from aerobic glycolysis is unfavorable for a response to therapy. There are a variety of ways to make cold tumors hot. These include injecting tumors with virus, cytokines, TLR agonist or using engineered T cells or chimeric antigen receptor T cells. Combining anti PD1 therapy with anti-CTLA4 therapy which recruits T cells from the periphery may also be another approach. Other approaches include combining anti-PD1 therapy with something which generates cytotoxic cell death (such as chemotherapy or radiation) or combining anti-PD1 therapy with targeted therapies that remodel the tumor microenvironment such as PI3K inhibitors, mTOR inhibitors, cyclin-dependent kinase inhibitors. The last approach seems to be the most logical because HCC has shown efficacy with drugs such as everolimus (mTOR inhibitor) and the multikinase inhibitor Lenvatinib. Indeed in other tumors such as kidney cancer and melanoma resistant to monotherapy with pembrolizumab, the combination of lenvatinib with pembrolizumab has shown response (56). Therefore in HCC, combination therapy will be required for efficacy.

Overall, our findings are novel reporting for the first time the immune landscape of HCC. Our findings reveal possible new combination immunotherapy approaches that may be exploited to treat these cancers.

## Supplementary Material

Figure S1 Patient, tumor, and treatment characteristics are shown in A. Kaplan–Meier plots of locoregional and distant recurrence-free survival are shown in B and C, respectively.

Figure S2 The most frequently mutated genes included MADCAM1 (12%), EIF1AX (10%), NF1 (10%), PTPRS (10%), NRAS (8%), TP53 (8%) (A). The majority of mutations were SNVs with a small percentage of deletions and insertions (B). Missense mutations were most common followed by splice site mutations, frameshift deletions and nonsense mutations. The most common DNA substitution types were C>T transitions, followed by T>C transitions, C>A transversions, C>G transversions. The distribution of DNA substitution type per tumor is shown in C.

Figure S3 Scatter plot matrix showing the negative correlation between SNV mutations, neoantigen load, and immune infiltration scores.

Figure S4 RNA-seq Immune infiltration stratified by LOH.

Table S1 Immune staining in HCC tumors

Table S2 Somatic mutations, insertions, deletions identified in 40 HCC tumors

Table S3 Neopeptides (8mer) associated with SNV alterations in HCC

Table S4 Neopeptides (9mer) associated with SNV alterations in HCC

Table S5 Neopeptides (10mer) associated with SNV alterations in HCC

Table S6 Neopeptides (11mer) associated with SNV alterations in HCC

Table S7 Neopeptides (8mer) associated with INDELS in HCC

Table S8 Neopeptides (9mer) associated with INDELS in HCC

Table S9 Neopeptides (10mer) associated with INDELS in HCC

Table S10 Neopeptides (11mer) associated with INDELS in HCC

Table S11 HLA divergence score overall and for each HLA type stratified by recurrence and phenotype

Table S12 HED results
