# Supplementary material for: Characterizing the Immune Microenvironment and Neoantigen Landscape of Hürthle Cell Carcinoma to Identify Potential Immunologic Vulnerabilities
Source: Cancer Res Commun. 2023 Jul 31;3(7):1409–22. doi: 10.1158/2767-9764.CRC-23-0120 (PMC10389111; doi:10.1158/2767-9764.CRC-23-0120)
Supplement: Figure S1 — Patient, tumor, and treatment characteristics are shown in A. Kaplan–Meier plots of locoregional and distant recurrence-free survival are shown in B and C, respectively. [file crc-23-0120-s01.pdf]

Figure S1

A

Table 1: Patient, tumor and treatment characteristics for minimally and widely invasive HCC (n=40*)

Variable	Minimally Invasive	%	Widely Invasive	%	p value
**Age at diagnosis**					
<45y	4	24%	3	14%	
≥45y	13	76%	18	86%	0.46
**Gender**					
Male	9	53%	7	33%	
Female	8	47%	14	67%	0.22
**Surgery**					
Lobectomy	4	24%	2	10%	
Total thyroidectomy	13	76%	19	90%	0.24
**RAI**					
No	12	70%	4	19%	
Yes	5	30%	17	81%	0.001
**pT Stage**					
T1	2	12%	0	0%	
T2	10	59%	9	42%	
T3	5	29%	6	28%	
T4	0	0%	6	28%	0.016
**pN Stage**					
N0Nx	17	100%	17	80%	
N1a	0	0%	2	10%	
N1b	0	0%	2	10%	0.14
**Overall stage**					
I	6	35%	2	10%	
II	7	41%	8	38%	
III	4	24%	5	24%	
IV	0	0%	6	28%	0.002

*Clinical data on 2 patients was not available.

B

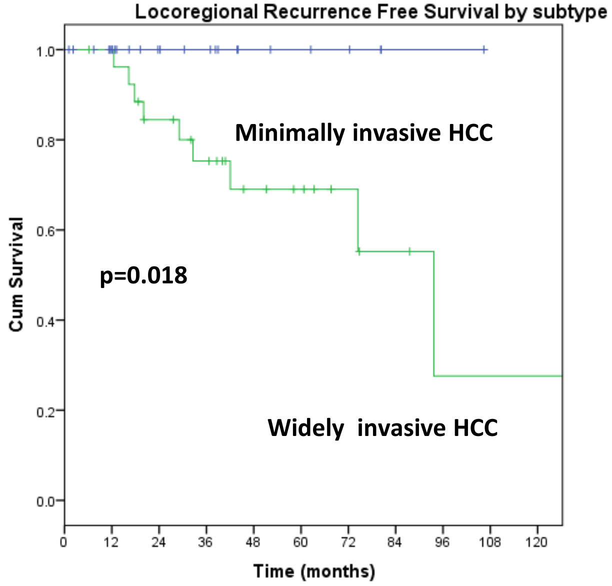

C

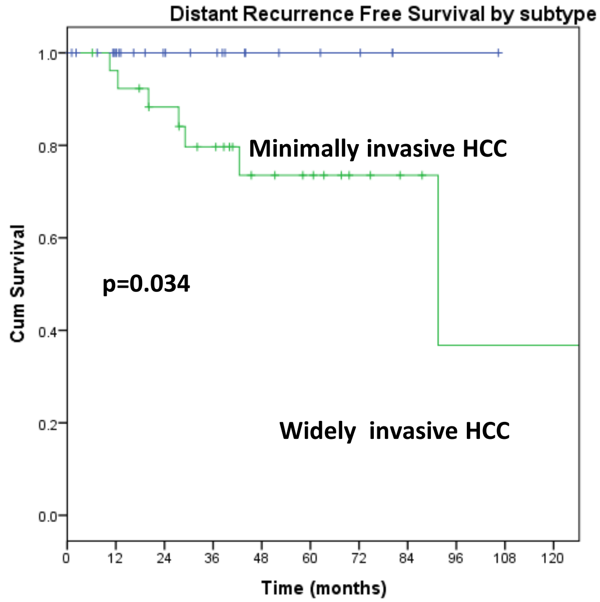

Figure S1. Patient, tumor, and treatment characteristics are shown in A. Kaplan–Meier plots of locoregional and distant recurrence-free survival are shown in B and C, respectively.
