# Supplementary material for: Characterizing the Immune Microenvironment and Neoantigen Landscape of Hürthle Cell Carcinoma to Identify Potential Immunologic Vulnerabilities
Source: Cancer Res Commun. 2023 Jul 31;3(7):1409–22. doi: 10.1158/2767-9764.CRC-23-0120 (PMC10389111; doi:10.1158/2767-9764.CRC-23-0120)
Supplement: Figure S2 — The most frequently mutated genes included MADCAM1 (12%), EIF1AX (10%), NF1 (10%), PTPRS (10%), NRAS (8%), TP53 (8%) (A). The majority of mutations were SNVs with a small percentage of deletions and insertions (B). Missense mutations were most common followed by splice site mutations, frameshift deletions and nonsense mutations. The most common DNA substitution types were C>T transitions, followed by T>C transitions, C>A transversions, C>G transversions. The distribution of DNA substitution type per tumor is shown in C. [file crc-23-0120-s02.pdf]

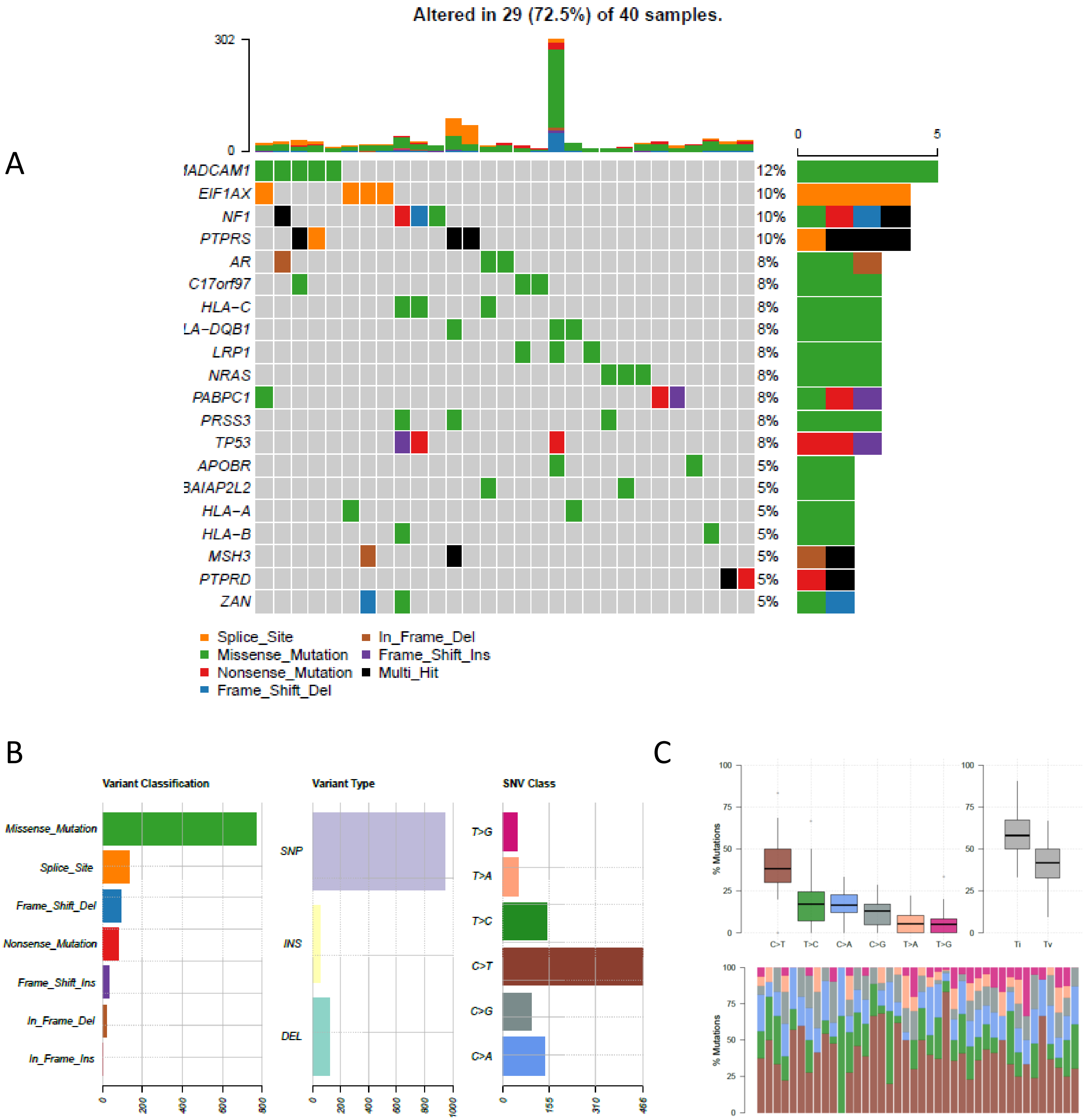

Figure S2. The most frequently mutated genes included MADCAM1 (12%), EIF1AX (10%), NF1 (10%), PTPRS (10%), NRAS (8%), TP53 (8%) (A). The majority of mutations were SNVs with a small percentage of deletions and insertions (B). Missense mutations were most common followed by splice site mutations, frameshift deletions and nonsense mutations. The most common DNA substitution types were C>T transitions, followed by T>C transitions, C>A transversions, C>G transversions. The distribution of DNA substitution type per tumor is shown in C.

Figure S2
