# Supplementary material for: Characterizing the Immune Microenvironment and Neoantigen Landscape of Hürthle Cell Carcinoma to Identify Potential Immunologic Vulnerabilities
Source: Cancer Res Commun. 2023 Jul 31;3(7):1409–22. doi: 10.1158/2767-9764.CRC-23-0120 (PMC10389111; doi:10.1158/2767-9764.CRC-23-0120)
Supplement: Figure S3 — Scatter plot matrix showing the negative correlation between SNV mutations, neoantigen load, and immune infiltration scores. [file crc-23-0120-s03.pdf]

SNV mutations and neoantigen correlation with immune infiltration scores

Spearman Correlation Coefficients<br>Prob > |r| under H0: Rho=0<br>Number of Observations	CYT	StromalScore	ImmuneScore	ESTIMATEScore	IIS	TIS
**SNVs**<br>SNVs	-0.19563<br>0.2675<br>34	0.00552<br>0.9753<br>34	-0.06833<br>0.7010<br>34	-0.06986<br>0.6946<br>34	-0.06189<br>0.7280<br>34	-0.30088<br>0.0838<br>34
**SNV_Total_NACnt**<br>SNV Total NACnt	-0.19291<br>0.2744<br>34	-0.16876<br>0.3400<br>34	-0.17013<br>0.3361<br>34	-0.21385<br>0.2246<br>34	-0.16906<br>0.3392<br>34	-0.39376<br>0.0212<br>34
**INDELs**<br>INDELs	-0.23059<br>0.2675<br>25	0.27899<br>0.1769<br>25	0.10191<br>0.6279<br>25	0.17392<br>0.4057<br>25	-0.09877<br>0.6386<br>25	-0.30811<br>0.1340<br>25
**IND_Total_NACnt**<br>IND Total NACnt	-0.29077<br>0.1585<br>25	-0.00544<br>0.9794<br>25	-0.04076<br>0.8466<br>25	-0.08735<br>0.6780<br>25	-0.23099<br>0.2666<br>25	-0.19139<br>0.3594<br>25

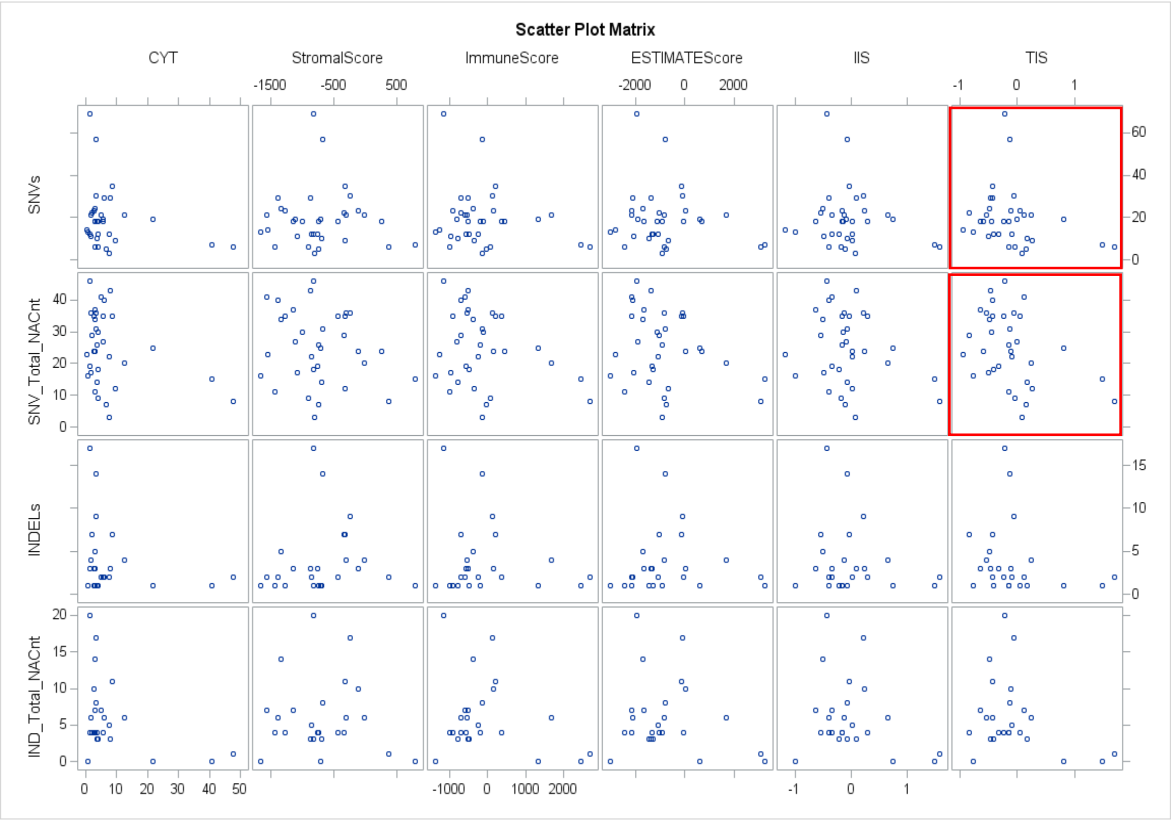

Figure S3. Scatter plot matrix showing the negative correlation between SNV mutations, neoantigen load, and immune infiltration scores.

Figure S3.
