# Supplementary material for: Characterizing the Immune Microenvironment and Neoantigen Landscape of Hürthle Cell Carcinoma to Identify Potential Immunologic Vulnerabilities
Source: Cancer Res Commun. 2023 Jul 31;3(7):1409–22. doi: 10.1158/2767-9764.CRC-23-0120 (PMC10389111; doi:10.1158/2767-9764.CRC-23-0120)
Supplement: Figure S4 — RNA-seq Immune infiltration stratified by LOH. [file crc-23-0120-s04.pdf]

RNASeq Immune infiltration stratified by LOH

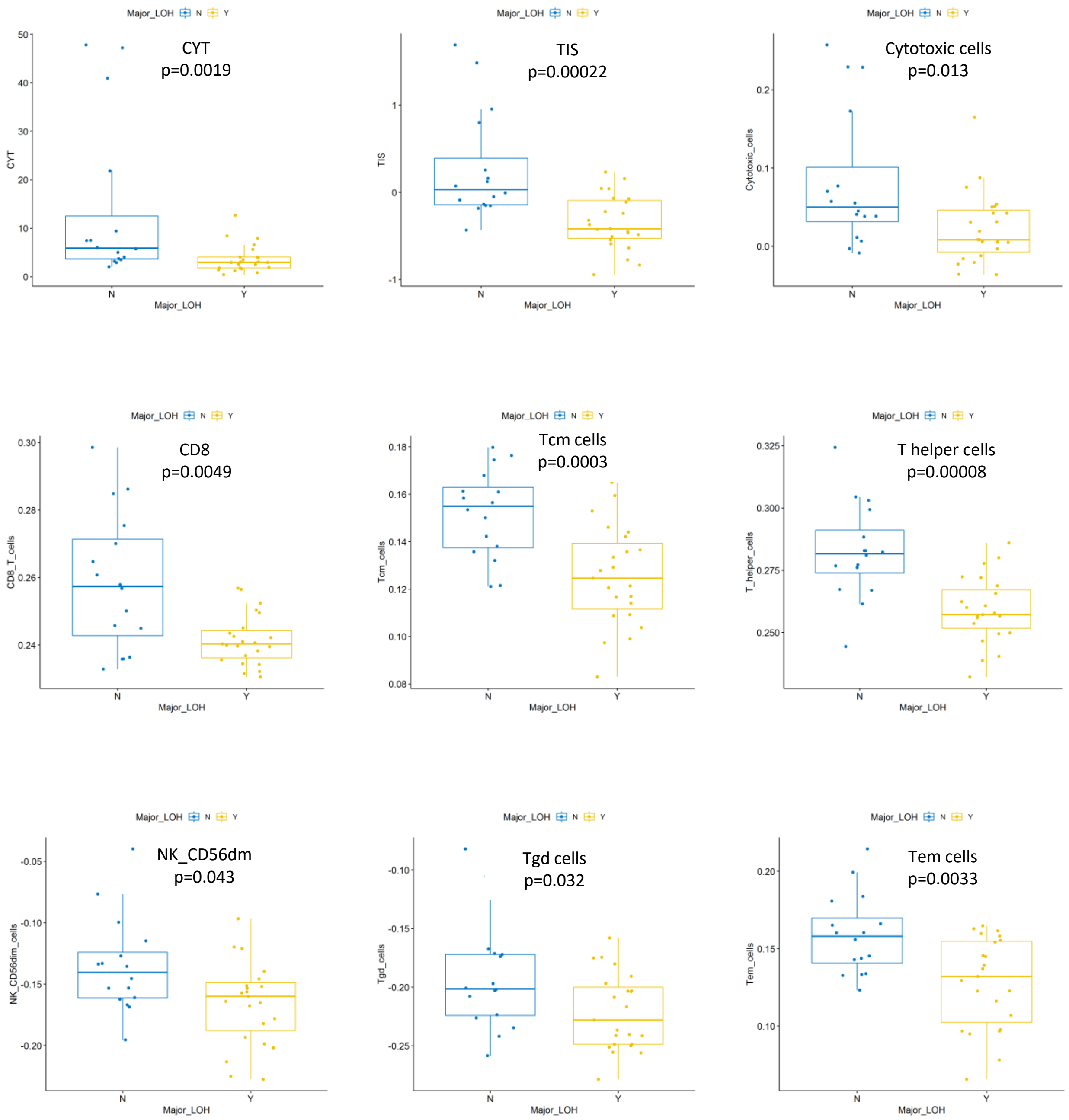

Figure S4. RNA-seq Immune infiltration stratified by LOH.

Figure S4
