# Supplementary material for: Characterizing the Immune Microenvironment and Neoantigen Landscape of Hürthle Cell Carcinoma to Identify Potential Immunologic Vulnerabilities
Source: Cancer Res Commun. 2023 Jul 31;3(7):1409–22. doi: 10.1158/2767-9764.CRC-23-0120 (PMC10389111; doi:10.1158/2767-9764.CRC-23-0120)

|  | HLA A divergence | HLA B divergence | HLA C divergence | Mean HLA divergence |
| --- | --- | --- | --- | --- |
| Group 1 vs. Group 2 | 0.4785 | 1 | 0.0172 | 0.694 |
| Group 1 vs. Group 3 | 0.2047 | 0.8504 | 0.4998 | 0.6723 |

Table S12. HED results

*Number of patients each group: Group 1 (9), Group 2 (20), Group 3 (17). p-values from Wilcoxon rank-sum test with continuity correction.

| Gp1=HWIDE recurrent |
| --- |
| Gp2=HWIDE nonrecurrent |
| Gp3=HMIN nonrecurrent |


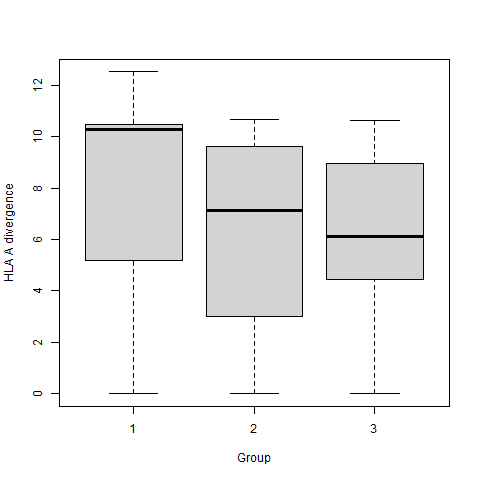

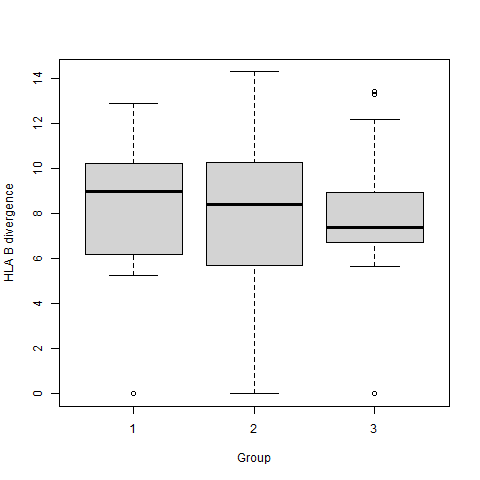

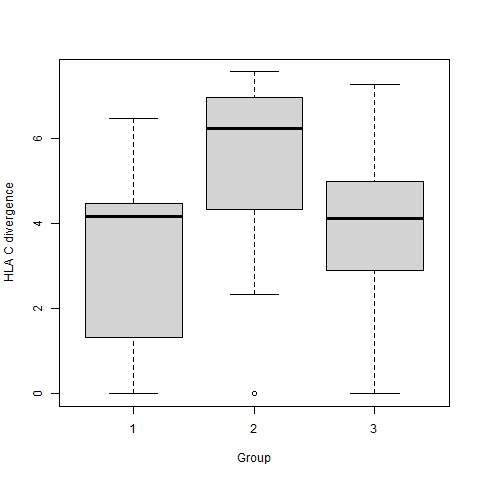

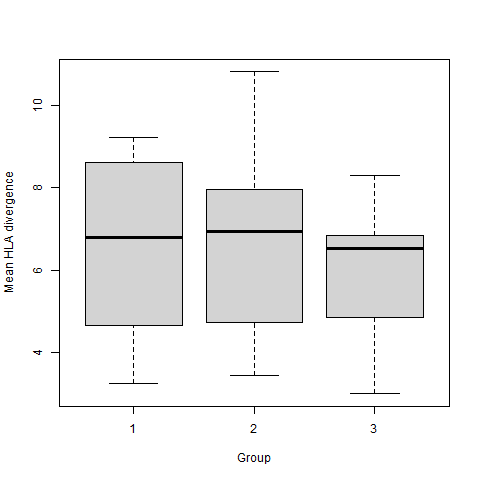

Supplement: Table S12 — HED results [file crc-23-0120-s16.docx]
